# Racial disparity in microvascular function among non‐Hispanic white and non‐Hispanic black men with newly diagnosed prostate cancer

**DOI:** 10.14814/phy2.70834

**Published:** 2026-04-17

**Authors:** Abigayle B. Simon, Jacob C. Looney, Marsha Blackburn, Reva Crandall, Jeffrey Thomas, Avirup Guha, Martha K. Terris, Zachary Klaassen, Neal L. Weintraub, Paula Rodriguez‐Miguelez, Ryan A. Harris

**Affiliations:** ^1^ Georgia Prevention Institute, Medical College of Georgia Augusta University Augusta Georgia USA; ^2^ Department of Cardiology, Medical College of Georgia Augusta University Augusta Georgia USA; ^3^ Department of Urology, Medical College of Georgia Augusta University Augusta Georgia USA; ^4^ Department of Kinesiology and Health Sciences Virginia Commonwealth University Richmond Virginia USA; ^5^ Division of Pulmonary and Critical Care, Department of Internal Medicine Virginia Commonwealth University Richmond Virginia USA

**Keywords:** arterial stiffness, endothelial function, race, vascular function

## Abstract

Cardiovascular disease (CVD) is a leading cause of mortality in men with prostate cancer (PC), with non‐Hispanic Black (NHB) men experiencing disproportionately higher CVD‐related mortality compared to non‐Hispanic White (NHW) men. Vascular endothelial dysfunction precedes overt CVD; however, the mechanisms underlying racial disparities in CVD among men with PC remain unclear. This study tested the hypothesis that NHB men with PC would exhibit impaired vascular health compared to NHW men. Thirty‐four men (12 NHW and 22 NHB) with a clinical diagnosis of PC underwent comprehensive vascular assessment, including arterial stiffness (pulse wave velocity [PWV] and augmentation index at 75 bpm [AIx75]), conduit‐vessel endothelial function (flow‐mediated dilation [FMD]), and microvascular function (post‐occlusive reactive hyperemia [PORH], local thermal heating [LTH], and acetylcholine iontophoresis). Clinical laboratory values, senescence‐associated secretory phenotype (SASP), and allostatic load were also evaluated. NHW men were older (*p* = 0.050), with no differences in body mass index (BMI), laboratory values, allostatic load, or SASP (all *p* > 0.05). NHB men demonstrated significantly lower PORH, LTH, and acetylcholine responses (all *p* < 0.001), while PWV, AIx75, and FMD were similar between groups. Despite comparable conduit‐vessel and arterial stiffness measures, NHB men exhibited impaired microvascular function, suggesting racial differences in vascular bed‐specific dysfunction following PC diagnosis.

## Graphical abstract

In newly diagnosed prostate cancer, non‐Hispanic Black men exhibit greater microvascular impairment than non‐Hipanic White men, which may increase cardiovascular disease risk.

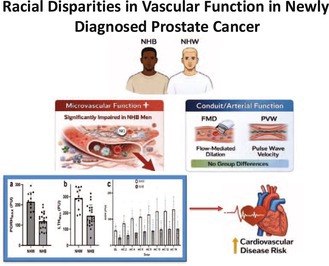

## 1 INTRODUCTION

Cardiovascular disease (CVD) is the leading cause of death worldwide, followed by cancer. Specifically, prostate cancer is the most prevalent type of cancer in men (Jain et al., 2024), and CVD is the leading cause of mortality, accounting for 31% of non‐cancer‐related deaths in men with prostate cancer (Van Hemelrijck et al., 2016). In fact, over the 10‐year period after prostate cancer diagnosis, CVD accounts for 32% of deaths in men with prostate cancer, whereas cancer itself accounts for 20% (Sturgeon et al., 2019). Indeed, vascular aging involves alterations in the mechanical and structural properties of blood vessels, changes that are commonly associated with the natural aging process and contribute to the pathogenesis of atherosclerosis and overt CVD (Ungvari et al., 2018). However, evidence suggests that the onset of cancer itself can act as a catalyst, accelerating these age‐related vascular impairments (Bhatia et al., 2022; Campisi, 2013). This may occur through mechanisms such as systemic inflammation, oxidative stress, and paraneoplastic effects that alter vascular homeostasis (Riscuta, 2016). In the case of prostate cancer, tumor‐induced biological changes, such as increased inflammatory cytokines, hormonal imbalances, and endothelial dysfunction, may further disrupt vascular integrity, compounding the risk of future cardiovascular events (Terwoord et al., 2022).

Vascular endothelial dysfunction plays a pivotal role in the etiology and progression of atherosclerotic CVD. In fact, conduit‐ and microvascular‐endothelial dysfunction and arterial stiffness are independent risk factors of CVD (Widmer & Lerman, 2014) and have been shown to predict future cardiovascular disease and events (Maruhashi et al., 2020; Yeboah et al., 2009). Microvascular dysfunction is not only representative of early vascular issues (Katunaric et al., 2021), but also sets the stage for broader cardiovascular complications that can be exacerbated by cancer progression. In addition, CVD risk prediction tools, such as the Framingham Risk Score (Lloyd‐Jones et al., 2004), are widely used in clinical practice to predict future cardiovascular events and can provide additional prognostic value in CVD risk assessment.

Racial disparities in cancer outcomes are well‐documented, particularly in prostate cancer, where significant differences in diagnosis and prognosis exist between racial groups. Specifically, non‐Hispanic Black (NHB) individuals generally present at diagnosis with a later stage and more aggressive form of cancer compared to their non‐Hispanic White (NHW) counterparts (Bandera et al., 2016; Noel & Fiscella, 2019). In addition, NHB men with prostate cancer have a four times higher risk of heart disease (Haque et al., 2017) and a 134% higher rate of mortality compared to NHW men (Rebbeck, 2018). Race differences in vascular health outcomes are important to consider, as disparities in cardiovascular health may affect cancer progression and overall health in men with prostate cancer (Dee et al., 2024; Kunhiraman & Guha, 2024; Troeschel et al., 2020). Although social determinants of health (SDOH) have been proposed to play a role (Powell‐Wiley et al., 2022), the underlying mechanisms that contribute to the race‐related health disparities in CVD risk have yet to be fully understood. Given emerging evidence suggesting that cancer‐related physiological stress (Vignjević Petrinović et al., 2023) and systemic inflammation (Libby & Kobold, 2019) may acutely affect vascular function, elucidating the impact that a recent diagnosis of prostate cancer has on different vascular beds may provide mechanistic insight into the pathways contributing to the accelerated and disparate CVD risk observed in this population. Accordingly, the present investigation sought to comprehensively assess vascular health in men who were recently diagnosed with prostate cancer and determine if race differences exist. It was hypothesized that NHB men with prostate cancer would exhibit higher arterial stiffness and lower conduit‐ and microvascular function when compared to NHW men with prostate cancer.

## 2 METHODS

### 2.1 Experimental design

All participants reported to the Georgia Prevention Institute at Augusta University following an overnight fast and having abstained from caffeine, smoking or any tobacco use, or moderate‐to‐vigorous exercise for 12 h prior to testing. The visit consisted of a written informed consent process, body composition measurements, anthropometric measures, and a comprehensive evaluation of vascular health, which was comprised of arterial stiffness, conduit‐vessel endothelial function, and microvascular function. Height and body mass were determined using a stadiometer and standard platform scale (CN20, DETECTO, Webb City, MO), respectively, and were used to calculate body mass index (BMI). Total body fat and lean mass were determined using dual energy X‐ray absorptiometry (QDR‐4500 W; Hologic, Waltham, MA). A single stick blood draw was performed to obtain clinical laboratory values and evaluate the Senescence‐Associated Secretory Phenotype (SASP). The Framingham Risk Score (FRS), indices of social determinants of health (SDOH), and daily physical activity was also assessed as an index of lifestyle behavior.

### 2.2 Participants

Thirty‐four men (NHW: *n* = 12; NHB: *n* = 22) with a diagnosis of prostate cancer were recruited to participate in the study. Eligibility criteria included a confirmed diagnosis of American Joint Committee on Cancer (AJCC) stage 1–3 prostate cancer. Participants were required to be 18 years of age or older, and individuals of all racial backgrounds were eligible for inclusion. The racial distribution reflects the demographic characteristics of the geographic area in which the study was conducted. Participants were excluded if they (1) had a previous diagnosis of major cardiovascular disease (i.e., myocardial infarction, congestive heart failure, or stroke), (2) had AJCC stage 4 prostate cancer, (3) had a history of radiation therapy, or (4) had a life expectancy of less than 1 year. Although the present investigation aimed to determine the impact of race on vascular health in men with prostate cancer, we include comparison vascular health data from a cohort of apparently healthy, age‐matched control men (*n* = 8; NHW: *n* = 6; NHB: *n* = 2) derived from ancillary studies conducted by our team. Importantly, the control data were collected and analyzed following identical methods. All study protocols were approved by the Institutional Review Board at Augusta University and Virginia Commonwealth University. This study was conducted in compliance with the ethical standards of the responsible institution on human subjects as well as with the Helsinki Declaration.

### 2.3 Framingham risk score, social determinants of health, and daily physical activity

The FRS was used to assess 10‐year cardiovascular disease risk from the following patient characteristics: smoking status, age, lipid profile, sex, and blood pressure (Lloyd‐Jones et al., 2004). Social determinants of health were assessed using an index of allostatic load by evaluating positive indicators for lipid levels, glucose concentrations, waist circumference, and the use of medications for diabetes, hypertension, or hypercholesterolemia as previously described (Xing et al., 2020). The Hospital Anxiety and Depression Scale (HADS) was also used to assess each participant's emotional status.

All participants were asked to wear triaxial accelerometers (ActiGraph Model GT3X+, ActiGraph, Pensacola, FL) on their non‐dominant wrist for 7 days while continuing with their normal activities. At the end of the 7‐day period, participants were instructed to return the accelerometer to the Georgia Prevention Institute by mail using a postage‐paid envelope. All indices of physical activity were derived by processing raw accelerometer data (.gt3x) that were collected at 30 Hz time resolution. Sedentary time is defined as the percentage of inactivity of total wearing time. Moderate‐vigorous physical activity (MVPA) was calculated using the Freedson Adult Equation (Freedson et al., 1998) and is reported as the total minutes per day.

### 2.4 Clinical laboratory values and senescence‐associated secretory phenotype

A venous blood sample was collected into serum separator or EDTA‐treated collection tubes (BD, United States) and serum or plasma, respectively, was separated out by centrifugation. Clinical laboratory values, including fasting blood glucose, fasting insulin, hemoglobin A_1c_, a complete lipid panel, and circulating testosterone, were determined using standard clinical core laboratory techniques (Laboratory Corporation of America Holdings, Burlington, NC).

The SASP, a panel of inflammatory cytokines linked to vascular aging, was assessed in serum and plasma samples that were flash frozen in liquid nitrogen and stored at − 80°C until later analyzed. Concentrations of plasma ET‐1, TNF‐α, IL‐6, IL‐18, IL‐15, CXCL‐10, IFN‐γ, IL‐8, GDF‐15, myeloperoxidase (MPO), osteopontin (OPN), Tumor Necrosis Factor‐Related Apoptosis Inducing Ligand (TRAIL), Procalcitonin, and Galectin‐3 were determined using a Simple Plex cartridge on the Ella Platform (Protein Simple, San Jose, CA) according to the manufacturer's instructions. The limits of detection for the measured human biomarkers were as follows: ET‐1 (0.250–1000 pg/mL), TNF‐α (1.14–290 pg/mL), IL‐6 (0.41–3850 pg/mL), IL‐18 (0.96–3660 pg/mL), IL‐15 (0.51–1950 pg/mL), CXCL‐10 (0.6–920 pg/mL), IFN‐γ (0.17–4000 pg/mL), IL‐8 (0.19–1804 pg/mL), GDF‐15 (0.52–2000 pg/mL), MPO (3.14–30,000 pg/mL), OPN (20.97–12,800 pg/mL), TRAIL (2.62–4000 pg/mL), procalcitonin (1.58–15,100 pg/mL), and galectin‐3 (3.11–4740 pg/mL).

### 2.5 Arterial stiffness

Arterial stiffness was determined noninvasively using the SphygmoCor XCEL system (AtCor Medical, Sydney, Australia). Briefly, pulse wave analysis (PWA) includes assessment of augmentation index (AIx) from the left brachial artery in duplicate, which was normalized for a heart rate of 75 beats/min (AIx75). The average reading was used for analysis. In addition, augmented aortic pressure (aAP) was measured, and the subendocardial viability ratio (SEVR) was also evaluated, which is an index of myocardial oxygen supply and demand (Tsiachris et al., 2012). Finally, carotid‐femoral pulse wave velocity (PWV) was determined by simultaneously recording electrocardiographic‐gated carotid and femoral artery waveforms and assessing the distance from each site to calculate a velocity, as previously described by our group (Rodriguez‐Miguelez et al., 2015).

### 2.6 Conduit‐vessel endothelial function

Conduit‐vessel function was assessed using brachial artery flow‐mediated dilation (FMD) in accordance with established guidelines (Harris et al., 2010). After a 15‐min supine rest, baseline brachial artery images and velocity profiles were recorded using a 12 MHz linear transducer (Logiq 7, GE Medical Systems). A forearm cuff was inflated to 250 mm Hg for 5 min, then released to induce reactive hyperemia. Diameter and velocity were recorded continuously for 2 min. End‐diastolic diameters were R‐wave gated and analyzed offline (Medical Imaging Applications). FMD (%) was calculated as the percent change from baseline diameter. Cumulative shear rate (AUC) was determined using the trapezoidal rule, and time to peak dilation (TTP) was recorded.

### 2.7 Microvascular function testing

Cutaneous microvascular blood flow was assessed using Laser Doppler Imaging (Moor FLPI‐2; Moor Instruments), a technique previously described by our group (Rodriguez‐Miguelez et al., 2016) and others (Roustit & Cracowski, 2012; Roustit & Cracowski, 2013). This method is minimally influenced by skin pigmentation, supporting its reliability in racially diverse populations (Abdulhameed et al., 2019). Participants rested supine for 15 min before testing. A forearm cuff was placed distal to the medial epicondyle. A round heater probe and iontophoresis chamber were secured on the ventral forearm using double‐sided adhesive tape, avoiding areas with tattoos, broken skin, or visible veins. Microvascular function was assessed using three protocols:

#### 2.7.1 Post‐occlusive reactive hyperemia (PORH)

The cuff was inflated to 250 mmHg for 5 min. Peak flux (PORH_MAX_), baseline flux (PORH_BL_), and time to peak (PORH_TTP_) were recorded.

#### 2.7.2 Local thermal hyperemia (LTH)

A heating probe raised the skin temperature to 44°C over 25 min. Peak flux (LTH_MAX_), baseline (LTH_BL_), and time to peak (LTH_TTP_) were recorded.

#### 2.7.3 Iontophoresis of acetylcholine (ACH)

A 2% ACH solution was delivered via iontophoresis (100 μA for 20 s, repeated seven times with 60 s intervals). Peak response (ACH_MAX_), baseline (ACH_BL_), and time to peak (ACH_TTP_) were measured.

Baseline flux for each protocol was calculated as the average of a 30‐s period prior to the intervention. PORH was always performed first, followed by LTH and then ACH iontophoresis, ensuring baseline recovery between tests. All responses were reported as maximal red blood cell flux (RBF) in perfusion units (PU). Cutaneous vascular conductance (CVC) was calculated as red blood cell flux (PU) divided by mean arterial pressure (MAP).

### 2.8 Statistical analyses

All data analyses were performed using SPSS Statistics Version 29. Independent samples *t*‐tests were performed to identify group differences (i.e., NHW vs. NHB; prostate cancer vs. controls). A 2 × 8 (group × time) mixed factorial, repeated measures analysis of variance was performed to identify race differences in the ACH response over time. Analysis of covariance was used to identify group differences while controlling for demographic variables (i.e., age). Pearson's Correlations were used to assess relationships between age and vascular function outcomes. Effect sizes are reported as Cohen's *d* values to represent small (Cohen's *d* = 0.2), medium (Cohen's *d* = 0.5), and large (Cohen's *d* = 0.8) effect sizes (Lakens, 2013). Data are reported as mean ± standard deviation (SD). Statistical significance (*) was set at *p* ≤ 0.05.

## 3 RESULTS

### 3.1 Participant characteristics, social determinants of health, and senescence‐associated secretory phenotype

Demographics and clinical laboratory values of men with prostate cancer are presented in Table 1. The average time since diagnosis to testing was 8 months (NHW: 7 ± 7 months; NHB: 10 ± 9 months; Cohen's *d* = 0.351; *p* = 0.373). NHB men with prostate cancer were 5 years younger (Cohen's *d* = −0.730; *p* = 0.050) compared with NHW men with prostate cancer. There was no difference in hypertension, use of hypertension medications, diabetes, smoking status or treatment status between NHW and NHB men (all *p* > 0.05). Daily physical activity, BMI, FRS, Gleason score and circulating total and free testosterone were all similar (all *p* > 0.05) between NHW and NHB men. No other differences in patient demographics or clinical laboratory values between NHB and NHW men with prostate cancer were observed (all *p* > 0.05). In addition, no differences in the HADS anxiety or HADS depression scores were observed between groups (all *p* > 0.05). No significant relationship among the FRS and tumor grade (*p* > 0.05) was observed. Further, no differences (all *p* > 0.05) in any SASP biomarkers between NHB and NHW men with prostate cancer were identified.

**TABLE 1:** Overall participant characteristics.

Variable	Overall	NHW	NHB	*p*‐value
	(*n* = 34)	(*n* = 12)	(*n* = 22)	
Demographic				
Age (years)	67 ± 7	70 ± 7	65 ± 7	**0.050**
Height (cm)	177 ± 7	179 ± 9	177 ± 7	0.389
Weight (kg)	90 ± 21	91 ± 25	90 ± 20	0.434
Hypertension (*n*)	26	8	18	0.167
Hypertension medication (*n*)	23	7	16	0.606
Diabetes (*n*)	10	4	6	0.721
Smoking (*n*)	15	3	12	0.169
Active surveillance/ADT (*n*)	24/10	10/2	14/8	0.241
BMI (kg/m^2^)	28.5 ± 6.1	28.3 ± 7.3	28.7 ± 5.5	0.874
Body fat (%)	32.8 ± 6.8	33.3 ± 6.3	32.6 ± 7.1	0.771
Lean mass (g)	53,667 ± 8649	52,178 ± 9271	54,412 ± 8445	0.493
VAT (g)	909 ± 391	990 ± 447	869 ± 364	0.410
MAP (mm Hg)	100 ± 10	96 ± 8	103 ± 10	0.073
Gleason Score	7 ± 1	7 ± 1	7 ± 1	0.472
Framingham Score (%)	16.5 ± 7.8	19.8 ± 11.4	15.2 ± 5.6	0.137
Sedentary time (%)	77 ± 11	79 ± 8	76 ± 12	0.397
MVPA (min/day)	90 ± 67	75 ± 42	98 ± 76	0.371
Social determinants of health				
Allostatic Load	3.1 ± 1.4	3.1 ± 1.6	3.1 ± 1.3	0.988
HADS—Anxiety	4.3 ± 3.5	4.6 ± 2.2	4.3 ± 4.0	0.845
HADS—Depression	3.1 ± 2.5	3.0 ± 1.8	3.2 ± 2.9	0.875
Clinical laboratory values				
HbA_1c_ (%)	6.7 ± 2.3	6.3 ± 0.9	7.1 ± 2.8	0.472
HOMA‐IR	1.8 ± 2.3	0.9 ± 0.5	2.3 ± 2.7	0.089
TC (mg/dL)	160 ± 40	147 ± 31	168 ± 43	0.148
HDL (mg/dL)	47 ± 19	39 ± 15	52 ± 20	0.059
LDL (mg/dL)	89 ± 32	90 ± 27	89 ± 36	0.953
TRIG (mg/dL)	97 ± 67	100 ± 41	96 ± 79	0.878
Testosterone, total (ng/dL)	307 ± 218	319 ± 174	301 ± 214	0.806
Testosterone, free (ng/dL)	33 ± 160	5 ± 2	50 ± 202	0.444
Senescence associated secretory phenotype				
ET‐1 (pg/mL)	2.4 ± 0.7	1.8 ± 0.5	2.6 ± 0.6	0.077
IL‐18 (pg/mL)	253 ± 94	287 ± 76	236 ± 100	0.252
IL‐15 (pg/mL)	4.3 ± 3.8	4.7 ± 4.7	4.2 ± 3.4	0.788
CXCL‐10 (pg/mL)	214 ± 156	160 ± 46	241 ± 185	0.275
IFN‐γ (pg/mL)	0.8 ± 0.9	0.8 ± 0.5	0.7 ± 1.0	0.825
TNF‐α (pg/mL)	10.8 ± 4.0	12.6 ± 4.6	9.9 ± 3.6	0.157
IL‐6 (pg/mL)	6.5 ± 10.8	6.4 ± 6.0	6.6 ± 12.7	0.969
IL‐8 (pg/mL)	8.4 ± 2.4	7.6 ± 3.1	8.8 ± 1.9	0.288
GDF‐15 (pg/mL)	1406 ± 790	1331 ± 431	1444 ± 926	0.706
MPO (pg/mL)	9694 ± 8980	9053 ± 5827	10,014 ± 10,315	0.777
OPN (ng/mL)	164 ± 163	139 ± 46	176 ± 197	0.566
TRAIL (pg/mL)	68 ± 34	63 ± 18	71 ± 40	0.516
Procalcitonin (pg/mL)	96 ± 45	96 ± 30	96 ± 51	0.991
Galectin‐3 (ng/mL)	9.1 ± 1.9	8.9 ± 2.0	9.2 ± 2.0	0.803

*Note*: Data are presented as mean ± SD; independent samples *t*‐test. Bold values indicate statistically significant.

Abbreviations: ADT, androgen deprivation therapy; BMI, body mass index; CXCL, chemokine ligand; ET, endothelin; GDF, growth‐derived factor; HADS, Hospital Anxiety and Depression Score; HbA_1c_, hemoglobin A_1c_; HDL, high‐density lipoprotein; HOMA‐IR, homeostatic model assessment for insulin resistance; IFN, interferon; IL, interleukin; LDL, low‐density lipoprotein; MAP, mean arterial pressure; MPO, myeloperoxidase; MVPA, moderate‐vigorous physical activity; OPN, osteopontin; TC, total cholesterol; TRAIL, tumor necrosis factor‐related apoptosis inducing ligand; TRIG, triglycerides; VAT, visceral adipose tissue.

Demographics of controls are presented in Table 2. There were no differences in age (*p* = 0.996), height (*p* = 0.257), weight (*p* = 0.671), or BMI (*p* = 0.475) between men with prostate cancer and controls.

**TABLE 2:** Differences in vascular function between men with prostate cancer vs. controls.

Variable	Prostate cancer	Healthy controls	*p*‐value
	(*n* = 34)	(*n* = 8)	
Age (years)	67 ± 7	67 ± 7	0.996
Height (cm)	177 ± 7	174 ± 4	0.257
Weight (kg)	90 ± 21	86 ± 21	0.671
BMI (kg/m^2^)	28.5 ± 6.1	28.7 ± 8.6	0.475
Arterial stiffness			
AIx75 (%)	21.7 ± 12.0	22.3 ± 9.8	0.909
PWV (m/s)	9.1 ± 1.2	6.7 ± 2.0	**<0.001**
Conduit‐vessel function			
FMD (%)	3.5 ± 2.9	7.0 ± 7.4	**0.035**
Local thermal heating (LTH)			
LTHMax (PU)	221 ± 84	286 ± 108	0.129
Post‐occlusive reactive hyperemia (PORH)			
PORHMax (PU)	153 ± 63	203 ± 86	0.099
Iontophoresis of acetylcholine (ACH)			
ACHMax (PU)	96 ± 51	117 ± 45	0.375

*Note*: Values are mean ± SD; independent samples *t*‐tests. Bold values indicate statistically significant.

Abbreviations: ACH, iontophoresis of acetylcholine; AIx75, augmentation index; BMI, body mass index; FMD, flow‐mediated dilation; LTH, local thermal heating; PORH, post‐occlusive reactive hyperemia; PWV, pulse wave velocity.

### 3.2 Microvascular function

**FIGURE 1:**
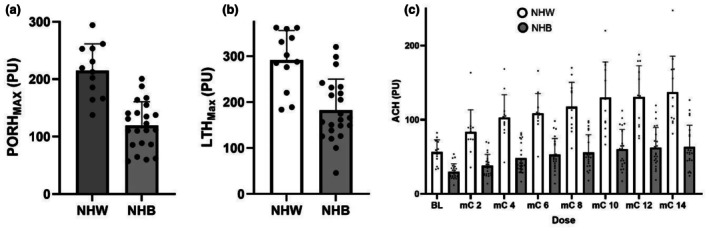
Differences in microvascular function between non‐Hispanic White (NHW) and non‐Hispanic Black (NHB) men with prostate cancer. (a) Post‐occlusive reactive hyperemia (PORH), (b) local thermal heating (LTH), (c) iontophoresis of acetylcholine (ACH) dose response; *n* = 34 (NHW = 12, NHB = 22). (a) and (b) Independent samples *t*‐test. (c) Repeated measures analysis of variance. Data are presented as mean ± SD. PU = perfusion units. *Indicates a significant difference from NHW.

The outcomes of the three microvascular function protocols are presented in Table 3. Vascular flux at baseline was significantly higher in NHW men during PORH (Cohen's *d* = −1.712; *p* < 0.001), LTH (Cohen's *d* = −1.188; *p* = 0.002), and ACH (Cohen's *d* = −1.874; *p* < 0.001) compared to NHB men. Additionally, the change from baseline was statistically lower in NHB men (PORH: Cohen's *d* = −1.126; *p* = 0.004; LTH: Cohen's *d* = −1.246; *p* < 0.001; ACH: Cohen's *d* = −1.103; *p* = 0.004). Further, the CVC was statistically lower in NHB men in PORH, LTH, and ACH (all *p* < 0.001). There were no other differences (all *p* > 0.05) in the outcomes of the microvascular function tests between NHW and NHB men with prostate cancer. Figure 1 illustrates the maximal cutaneous flux for PORH and LTH, and the ACH dose response protocol in NHB and NHW men. Specifically, PORH (Figure 1a; Cohen's *d* = −2.220; *p* < 0.001), LTH_MAX_ (Figure 1b; Cohen's *d* = −1.637; *p* < 0.001), and iontophoresis of acetylcholine (Figure 1c; Cohen's *d* at ACH_MAX_ = −2.085; *p* < 0.001) were lower in NHB men compared to NHW men. No significant correlations among the FRS and the assessments of microvascular function (all *p* > 0.05) were observed. No discomfort was reported from the participants during the heating process, which has also previously been reported with the same temperature (Roustit & Cracowski, 2012).

**TABLE 3:** Parameters of the FMD test and microvascular function testing.

Variable	NHW	NHB	*p*‐value
	(*n* = 12)	(*n* = 22)	
Arterial stiffness			
AIx75 (%)	21.0 ± 12.6	22.1 ± 12.0	0.798
aAP (mm Hg)	13.2 ± 7.5	13.0 ± 4.9	0.939
SEVR (%)	138 ± 27	153 ± 26	0.124
PWV (m/s)	9.2 ± 1.2	9.0 ± 1.3	0.689
Conduit‐vessel function			
FMD_BL_ (cm)	0.40 ± 0.05	0.42 ± 0.07	0.369
FMD_Peak_ (cm)	0.41 ± 0.05	0.43 ± 0.07	0.408
FMD_Shear_ (sec^−1^, AUC)	34,392 ± 14,119	30,723 ± 19,684	0.573
FMD/Shear	0.12 ± 0.10	0.13 ± 0.09	0.738
FMD_TTP_ (sec)	65.4 ± 19.6	57.5 ± 27.0	0.379
Local thermal heating (LTH)			
LTH_BL_ (PU)	65.8 ± 33.5	39.0 ± 15.6	**0.002**
LTH_TTP_ (sec)	15.7 ± 5.9	14.3 ± 6.7	0.525
Absolute change (PU)	226 ± 75	143 ± 61	**0.001**
CVC (PU/mm Hg)	3.1 ± 0.8	1.8 ± 0.7	**<0.001**
Post‐occlusive reactive hyperemia (PORH)			
PORH_BL_ (PU)	84.5 ± 41.1	39.1 ± 13.6	**<0.001**
PORH_TTP_ (sec)	15.9 ± 4.6	16.5 ± 7.4	0.771
PORH/LTH ratio	0.75 ± 0.14	0.70 ± 0.24	0.516
Absolute change (PU)	130 ± 61	80 ± 32	**0.004**
CVC (PU/mm Hg)	2.3 ± 0.6	1.2 ± 0.4	**<0.001**
Iontophoresis of acetylcholine (ACH)			
ACH_BL_ (PU)	73.5 ± 25.4	37.3 ± 15.2	**<0.001**
ACH_TTP_ (sec)	16.4 ± 4.7	15.5 ± 5.9	0.654
Absolute change (PU)	70 ± 49	30 ± 25	**0.004**
CVC (PU/mm Hg)	1.5 ± 0.5	0.7 ± 0.3	**<0.001**

*Note*: Values are mean ± SD; independent samples *t*‐tests. Bold values indicate statistically significant.

Abbreviations: aAP, Augmented aortic pressure; ACH, iontophoresis of acetylcholine; AIx75, augmentation index; BL, baseline; CVC, cutaneous vascular conductance; FMD, flow‐mediated dilation; LTH, local thermal heating, Absolute change, difference between peak flow and baseline flow; PORH, post‐occlusive reactive hyperemia; PWV, pulse wave velocity; SEVR, subendocardial viability ratio; TTP, time to peak.

Composite data from the microvascular function tests in men with prostate cancer and controls are presented in Table 2. There were no differences (all *p* > 0.05) in PORH_MAX_, LTH_MAX_, or ACH_MAX_ between patients and controls.

### 3.3 Conduit‐vessel function and arterial stiffness

**FIGURE 2:**
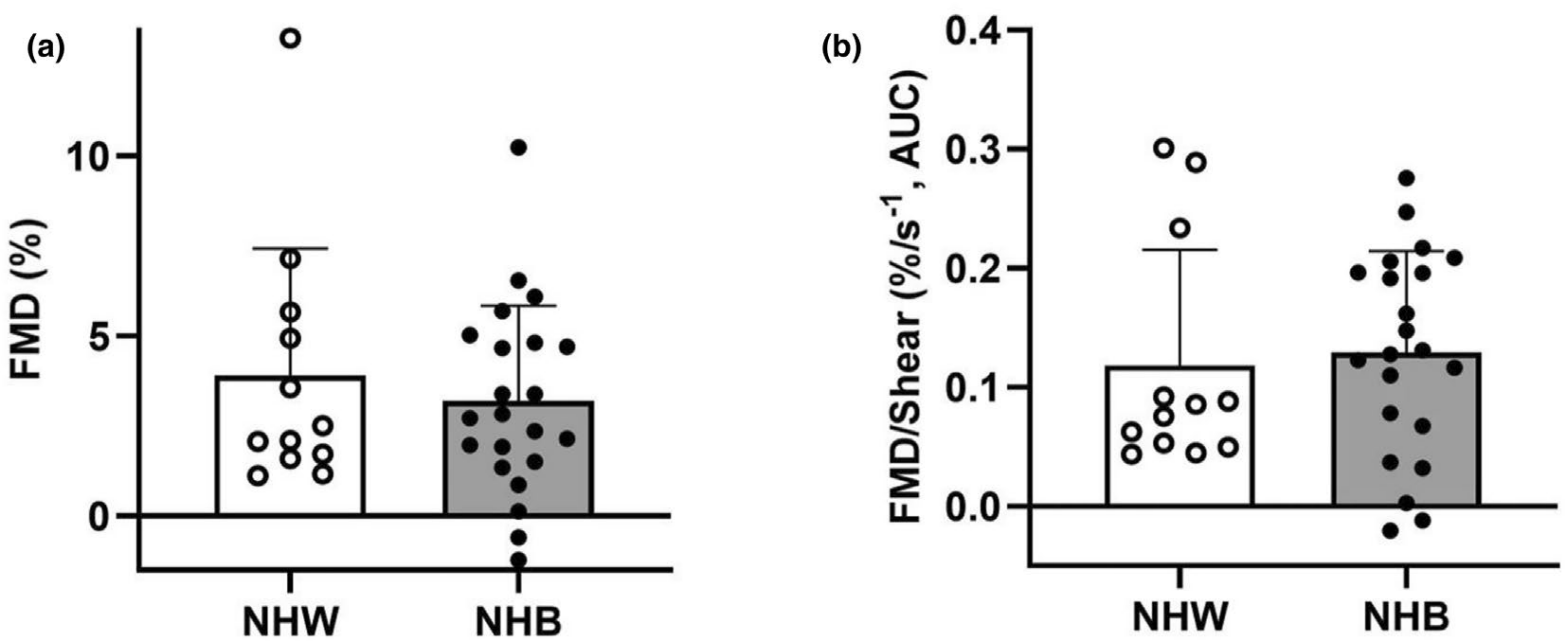
Differences in flow‐mediated dilation (FMD) between non‐Hispanic White (NHW) and non‐Hispanic Black (NHB) men with prostate cancer. (a) FMD, (b) FMD/Shear; *n* = 34 (NHW = 12, NHB = 22). Independent samples *t*‐test. Data are presented as mean ± SD.

The outcomes of the FMD test are presented in Table 3. There were no differences (all *p* > 0.05) in baseline diameter, peak diameter, shear, FMD normalized for shear, or FMD time to peak between NHW and NHB men. Figure 2 illustrates FMD in NHB and NHW men with prostate cancer. FMD was similar (Cohen's *d* = −0.236; *p* = 0.516) between groups. These findings persisted after controlling for age (Cohen's *d* = 0.390; *p* = 0.293). The difference in FMD between men with prostate cancer and controls is presented in Table 2. Controls exhibited a higher (*p* = 0.035) FMD compared to men with prostate cancer.

The outcomes of the arterial stiffness test are presented in Table 3. There were no differences (all *p* > 0.05) in AIx75, aAP, SEVR, or PWV between NHW and NHB men with prostate cancer. The findings persisted after controlling for age (all *p* > 0.05). The outcomes of the arterial stiffness test in men with prostate cancer and controls are presented in Table 2. Controls exhibited a lower (*p* < 0.001) PWV compared to men with prostate cancer; however, no difference in AIx75 (*p* > 0.05) between groups was observed.

## 4 DISCUSSION

Vascular endothelial dysfunction is an independent predictor of elevated CVD risk and cancer progression (Sedrak & Cohen, 2023) and plays a role in atherosclerotic CVD, the predominant cause of mortality among men with prostate cancer. Health disparities, particularly associated with race, further exacerbate the negative cardiovascular‐related outcomes in cancer. Accordingly, the overall findings from the present investigation demonstrate that NHB men with prostate cancer exhibit reduced microvascular function, despite being 5 years younger and exhibiting similar SDOH and SASP compared to NHW men with prostate cancer. In addition, no differences in arterial stiffness or conduit‐vessel endothelial function were observed between newly diagnosed NHW and NHB men with prostate cancer.

### 4.1 Prostate cancer and vascular health: Impact of race

#### 4.1.1 Microvascular function

In general, the microcirculation contributes to blood flow regulation and is responsible for the exchange of nutrients and gases at the cellular level. Microvascular dysfunction typically precedes the onset of both conduit‐vessel dysfunction and arterial stiffness, yet can independently predict the development of atherosclerosis (Katunaric et al., 2021). Findings of the present study demonstrate, for the first time, that microvascular function was lower in NHB men with recently diagnosed prostate cancer compared to NHW men.

All three indices of microvascular function assessed in the present investigation (PORH, LTH, and iontophoresis of acetylcholine) were lower in NHB men with prostate cancer, each of which interrogate different physiological mechanisms related to microvascular dysfunction. PORH reflects integrated microvascular reactivity influenced primarily by metabolic vasodilators and prostanoid pathways, with a relatively modest contribution from NO (Silber et al., 2007). In contrast, the plateau phase of LTH is predominantly NO‐dependent and serves as an established index of endothelial NO bioavailability within the cutaneous microcirculation (Choi et al., 2014). Acetylcholine iontophoresis elicits endothelium‐dependent vasodilation through muscarinic receptor activation and involves multiple signaling pathways, including NO, cyclooxygenase‐derived prostanoids, and endothelium‐derived hyperpolarizing factors (Kellogg Jr. et al., 2005). These three pathways provide mechanistic insight into CVD risk and identify potential therapeutic targets that may ultimately improve CVD outcomes in prostate cancer.

Baseline flux in all three protocols of microvascular testing was higher in NHW men compared with NHB men. It is plausible that prostate cancer treatment may influence cutaneous microvascular density differently in NHB and NHW men, thereby altering basal perfusion. Importantly, however, the absolute change from baseline for all three protocols was still statistically lower in NHB men, which suggests that the microvascular response observed was not solely attributable to differences in baseline cutaneous vascular flux.

Interestingly, although NHB men were significantly younger than NHW men, they demonstrated significantly lower microvascular function. Given that both groups were within an age range typically associated with vascular aging (Ungvari et al., 2018), this finding suggests that NHB men may experience earlier or more pronounced microvascular impairments compared to NHW men with prostate cancer. Nonetheless, the present findings align with prior evidence suggesting that NHB individuals may have heightened susceptibility to microvascular dysfunction and CVD (Bullock‐Palmer et al., 2024; Zhang et al., 2022), a disparity that may amplify cancer‐related vascular risk and contribute to worse cardiovascular outcomes over time.

An exploratory analysis incorporating an age‐matched apparently healthy male cohort was included to determine the combined influence of cancer and race on microvascular dysfunction. Although not the primary focus of the investigation, microvascular function was numerically higher (although not statistically significant) in male controls compared to men with prostate cancer; however, these differences did not reach statistical significance. In fact, controls exhibit the greatest microvascular responses, followed by NHW men with prostate cancer, and the lowest responses were observed in NHB men with prostate cancer. To further explore this pattern, an exploratory analysis comparing NHB men with prostate cancer directly to age‐matched healthy controls revealed significantly lower microvascular function across all testing modalities (PORH [*p* = 0.001], LTH [*p* = 0.005], and ACH [*p* = 0.002]), accompanied by lower FMD (*p* = 0.046), and greater PWV (*p* = 0.002), supporting a stepwise gradient of vascular impairment. An important consideration is whether the observed differences between healthy controls (predominantly NHW) and NHB men with prostate cancer are attributable to race, disease status, or an interaction between the two. While racial disparities in cardiovascular outcomes are well‐documented, our data suggest that the differences in microvascular function may not be explained by race alone, nor solely by the presence of prostate cancer. Rather, it is plausible that the interaction between race and a recent cancer diagnosis may differentially influence vascular physiology. Given the modest sample size and imbalance in racial representation among the control group, these findings should be interpreted with optimistic caution. Larger studies are needed to more definitively disentangle the independent and interactive contributions of race and disease status to vascular dysfunction in this population.

#### 4.1.2 Conduit‐vessel function and arterial stiffness

FMD is a non‐invasive marker of conduit‐vessel endothelial function and reflects nitric oxide bioavailability. FMD was similar between NHB and NHW men with prostate cancer, and this finding remained consistent after adjusting for age. This contrasts with the clear racial differences observed in microvascular function and suggests that racial disparities in vascular health may emerge first at the microvascular level before extending to the conduit vasculature. Perhaps unsurprisingly, FMD was significantly higher in controls compared to both NHB and NHW men with prostate cancer, indicating that prostate cancer itself, rather than race, is a key driver of early conduit‐vessel impairment. It is plausible that cancer‐related physiological stress may initiate early macrovascular dysfunction that does not yet differ by race at the time of diagnosis. Nonetheless, longitudinal evaluation of endothelial function is warranted to determine whether conduit‐vessel disparities develop over time and whether these changes contribute to downstream differences in cardiovascular risk.

Arterial stiffness is another critical indicator of cardiovascular health, closely linked with aging and risk for coronary heart disease and hypertension (Kim, 2023). No significant differences in arterial stiffness were observed between NHB and NHW men with prostate cancer, even after accounting for age. This mirrors findings from large population‐based cohorts showing minimal racial differences in arterial stiffness among otherwise healthy NHB and NHW adults (Morris et al., 2013). However, the present study extends this literature by examining an older population with a new cancer diagnosis, in whom vascular abnormalities might typically be more detectable. The absence of racial differences in conduit function and arterial stiffness, despite pronounced disparities in microvascular health, highlights a potential temporal progression in cancer‐related vascular injury: microvascular dysfunction may represent the earliest and most race‐sensitive marker, followed by more global macrovascular changes. However, these findings do not support a single, linear progression of vascular dysfunction, but instead suggest that the vascular bed most affected may depend on both disease status and race: comparisons with healthy controls indicate early cancer‐related impairments in conduit‐vessel function and arterial stiffness, whereas racial disparities within the prostate cancer cohort are most evident at the level of the microcirculation. Nonetheless, early detection and intervention aimed at preserving vascular compliance and endothelial function could therefore be valuable for reducing long‐term cardiovascular burden among men with newly diagnosed prostate cancer.

### 4.2 Factors related to CVD risk do not explain differences in microvascular function

The FRS, which estimates CVD risk based on factors like smoking, age, lipids, sex, and blood pressure, showed no differences between NHB and NHW men in this study. Physical activity, glycemic status, and lipid profiles were also similar. While FRS may not account well for racial disparities and its use in race‐based risk prediction is increasingly scrutinized (Obermeyer et al., 2019), no associations were found between FRS, microvascular function, or tumor grade (as indicated by Gleason score).

To assess social determinants of health (SDOH), allostatic load and HADS scores were compared. No race differences were observed. Although previous studies have reported higher allostatic load in NHB men with prostate cancer (Stabellini et al., 2022), different calculation methods may explain this discrepancy with the current findings. In addition, no significant racial differences were observed in the HADS, a validated tool for assessing anxiety and depression in patients with cancer (Annunziata et al., 2020). Collectively, these findings suggest that SDOH, as measured in the present investigation, may not fully account for disparities in microvascular function. Still, other unmeasured factors, such as socioeconomic status and healthcare access, could play a role and warrant further study.

Similarly, no racial differences were found in SASP biomarkers, a group of proteins reflecting cellular senescence due to physiological stress. These findings suggest that systemic inflammatory or senescent states likely do not explain the observed race differences in microvascular function. It is important to note, however, that changes in SASP over time could still contribute to progressive vascular dysfunction, a possibility that merits future research.

Nonetheless, the current study is the first to demonstrate that there are differences in microcirculation between NHB and NHW men with prostate cancer, albeit similar SDOH or SASP, suggesting that these disparities may stem from an interplay of racial background and prostate cancer rather than race alone. This underscores the urgent need for further research into the specific cardiovascular risks faced by NHB men with prostate cancer and suggests that early microvascular changes could accelerate vascular aging, emphasizing the importance of targeted early interventions.

### 4.3 Experimental considerations

Laser Doppler, unlike near‐infrared techniques, is generally considered to be minimally influenced by skin pigmentation and thus suitable for comparisons across diverse populations (Abdulhameed et al., 2019); however, prior validation studies were conducted in younger, healthy cohorts and may not fully generalize to older cancer populations. Thus, although unlikely, the potential influence of skin pigmentation, anatomical skin characteristics, and vitamin D status on vascular function independent of race or cancer status should be considered. Vitamin D deficiency, which is more prevalent among individuals with darker skin, has been associated with impaired endothelial function and may contribute to the observed differences. Importantly, absolute changes from baseline remained significantly lower in NHB men across all protocols, suggesting that the findings are not solely attributable to baseline perfusion differences or measurement artifact. Additionally, a limitation of the present study is the relatively small and unequal sample size, which may limit statistical power and generalizability; therefore, larger, adequately powered studies are needed to confirm these findings and further elucidate the mechanisms underlying racial differences in vascular function following prostate cancer diagnosis.

## 5 CONCLUSION

Findings of the present investigation identified that microvascular function is impaired in NHB men compared to NHW men with prostate cancer. Importantly, no differences in SDOH or SASP were observed between groups. Given that NHB men were 5 years younger and no differences in conduit‐vessel function and arterial stiffness were observed, these data provide evidence that microvascular dysfunction may be contributing to the racial disparity in CVD progression following prostate cancer diagnosis. In addition, the findings of the current study provide evidence that race can affect vascular beds differently following a recent diagnosis of prostate cancer. Indeed, understanding the potential mechanisms related to the health disparities in vascular function will help identify potential therapeutic targets that can reduce the cardiovascular disease burden associated with prostate cancer.

## AUTHOR CONTRIBUTIONS

A. B. S. contributed to data acquisition, data analysis and interpretation, statistical analysis, manuscript write‐up, manuscript review, and approved the final version. J. C. L. contributed to data analysis and interpretation, manuscript review, and approved the final version. M. B. contributed to data acquisition, manuscript review, and approved the final version. R. C. contributed to data acquisition, manuscript review, and approved the final version. J. T. contributed to data analysis, manuscript review, and approved the final version. A. G. contributed to data acquisition, manuscript review, and approved the final version. M. T. contributed to manuscript review and approved the final version. Z. K. contributed to manuscript review and approved the final version. R. L. W. contributed to data acquisition, manuscript review, and approved the final version. P. R. M. contributed to data acquisition, data analysis and interpretation, manuscript review, and approved the final version. R. A. H. contributed to study conception and design, data acquisition, data analysis and interpretation, manuscript review, and approved the final version.

## FUNDING INFORMATION

This work was supported by American Heart Association SFRN 863621 (RAH, AG, and NLW) and American Heart Association 18CDA34110323 (PRM).

## CONFLICT OF INTEREST STATEMENT

The authors have no conflicts of interest to disclose.

## ETHICS STATEMENT

Research was performed in accordance with the Declaration of Helsinki.

## Data Availability

The data that support the findings of this study are available on request from the corresponding author.
